# Graph retrieval augmented large language models for facial phenotype associated rare genetic disease

**DOI:** 10.1038/s41746-025-01955-x

**Published:** 2025-08-24

**Authors:** Jie Song, Zhichuan Xu, Mengqiao He, Jinhua Feng, Bairong Shen

**Affiliations:** 1 Department of Ophthalmology and Institutes for Systems Genetics, Frontiers Science Center for Disease-related Molecular Network, West China Hospital, Sichuan University, Chengdu, China; 2 School of Computer Science and Software Engineering, Southwest Petroleum University, Chengdu, China

**Keywords:** Diagnosis, Literature mining, Machine learning

## Abstract

Many rare genetic diseases have recognizable facial phenotypes that serve as diagnostic clues. While Large Language Models (LLMs) have shown potential in healthcare, their application to rare genetic diseases still faces challenges like hallucination and limited domain knowledge. To address these challenges, Retrieval-Augmented Generation (RAG) is an effective method, while Knowledge Graphs (KGs) provide more accurate and reliable information. In this paper, we constructed a Facial Phenotype Knowledge Graph (FPKG) including 6143 nodes and 19,282 relations and incorporate RAG to alleviate the hallucination of LLMs and enhance their ability to answer rare genetic disease questions. We evaluated eight LLMs across four tasks: domain-specific QA, diagnostic tests, consistency evaluation, and temperature analysis. The results showed that our approach improves both diagnostic accuracy and response consistency. Notably, RAG reduces temperature-induced variability by 53.94%. This study demonstrates that LLMs can effectively incorporate domain-specific KGs to enhance accuracy, and consistency, thereby improving diagnostic decision-making.

## Introduction

Facial phenotype refers to the morphological features of the face, and many rare genetic diseases have recognizable facial features. For instance, in a family with Crouzon syndrome, there is a high degree of overlap between the facial features of the parents and children, all of whom share facial phenotypes such as mandibular prognathism, shallow orbits, proptosis, and exotropia^1^. Specific facial features play a crucial role in the diagnosis of clinical disorders.

Many AI models have made significant advances in facial phenotypes-associated rare genetic diseases^2–4^. However, the potential of LLMs in this area has not been explored. LLMs are typically defined as transformer^5^ based language models containing more than hundreds of billions of parameters, such as GPT-4^6^, Claude^7^, Gemini^8^, LLaMA^9^. LLMs have garnered significant interest due to their impressive comprehension, inference, and generative capabilities, and have begun to show great potential in the field of biomedical, particularly in the areas of medical diagnostics^10^, mental health^11^, biological materials^12^, hospital management^13^, and medical education^14^.

Although LLMs show potential in the medical field, their application in facial phenotypes still faces significant challenges.

- **Hallucination**^15^**:** LLMs sometimes generate information that appears reasonable but is actually false or unfounded, which can lead to serious consequences in medical applications.
- **Lack of specialized domain knowledge**^16^**:** LLMs lack the ability to handle domain-specific and highly specialized queries. Although LLMs are excellent at understanding and generating natural language, they lack a deep understanding of facial phenotypes and related genetic diseases. This deficit in specialized knowledge can limit the effectiveness of LLMs in the field of facial phenotype.

Retrieval-Augmented Generation (RAG) is one of the approaches to address the above challenges^17^. Content generation is augmented by retrieving domain knowledge to reduce factual errors in knowledge-intensive tasks with the help of external knowledge. However, current RAG relies on dense vector similarity searches as a retrieval mechanism. This approach is insufficient for complex queries and may also encounter redundancy issues when similar information is retrieved from multiple sources, leading to duplicate responses^18,19^. The optimal RAG system will accurately retrieve only what is necessary, minimizing the inclusion of irrelevant information.

Meanwhile, Knowledge Graphs (KGs), which store structured knowledge in the form of triplet, i.e., [entity]-(relation)-[entity], provide structured and explicit representations of entities and relations, which are more accurate than retrieving information through vector similarity, provide higher quality context and facilitate the analysis of complex relations between entities, enabling us to search for “things not strings” by storing large numbers of explicit facts in the form of accurate, updatable and interpretable knowledge triplets^18–20^. Compared to generalized KGs, domain-specific KGs tend to be smaller in size but more accurate and reliable. The effectiveness of combining KG with LLMs has been demonstrated in other fields, such as RNA relation extraction^21^, knowledge discovery^22^, and materials design^23^. However, there is currently no KG for facial phenotype-associated rare genetic diseases.

Therefore, in this paper, we constructed a facial phenotype domain KG, called FPKG, based on the Human Phenotype Ontology (HPO)^24^ and combined it with two types of RAG (Cypher RAG, Vector RAG) to address the issues of hallucination and inability to answer questions in specialized domains for LLMs. Four experiments were conducted: domain knowledge question answering, diagnostic tests, consistency evaluation, and temperature analysis in order to provide a comprehensive assessment of retrieval-augmented LLMs. In addition, we constructed three benchmark datasets for facial phenotype-associated rare genetic diseases to support the four experiments mentioned above. In domain-specific QA, we asked the LLMs domain-related questions and evaluated their responses against reference answers based on the HPO. In diagnostic tests and consistency evaluation, each question was repeated five times to calculate average accuracy and response consistency. In temperature analysis, we investigated the impact of different temperature parameters on LLMs’ performance.

In summary, our contributions can be summarized as follows:

- We constructed a KG for facial phenotype-associated rare genetic diseases, encompassing 6143 nodes and 19,282 relationships across six entity types (facial phenotype, genotype, disease, variation, patient, and article). By rigorously comparing the two retrieval approaches of structured query-based (Cypher) retrieval and similarity-based (vector) retrieval, we identified their performance gaps and complementary strengths.
- We developed three benchmark datasets and established a four-dimensional evaluation framework (domain knowledge QA, diagnostic tests, temperature sensitivity, and response consistency) to assess eight LLMs. Our results showed RAG LLMs consistently outperformed vanilla counterparts across all experiments.
- The experiments demonstrate that LLMs have the potential to incorporate domain-specific KGs, and that the structured and accurate information provided by KGs can effectively reduce LLMs hallucinations while improving their consistency and accuracy, as well as reducing temperature-induced variability.

## Results

To evaluate the performance of Graph RAG, we implemented two distinct retrieval approaches. The Cypher-based RAG leverages the capability of LLMs to generate structured graph queries, enabling precise retrieval of relevant subgraphs through Neo4j’s Cypher queries. In contrast, the Vector-based RAG employed a biomedical named entity recognition model and graph embedding algorithms to perform similarity-based retrieval, demonstrating enhanced robustness for LLMs with limited Cypher generation capabilities.

Three categories of LLMs—Cypher RAG LLMs, Vector RAG LLMs, and Vanilla LLMs—were systematically evaluated through four experiments: (1) Domain Knowledge QA: Evaluating comprehension of phenotype-gene-disease relationships using both model-based and rule-based metrics for quantitative assessment. (2) Diagnostic tests: A clinical case-based evaluation comparing LLMs’ performance on structured multiple-choice and open-ended diagnostic tasks. (3) Temperature Analysis: Testing LLMs performance across a temperature range of 0 to 1.0. (4) Consistency Evaluation: Evaluating response consistency by repeating queries five times. All experiments were conducted via API calls across 8 LLMs (GPT-3.5-turbo, GPT-4-turbo, GPT-4o, Claude-3-opus, Claude-3-sonnet, Claude-3-haiku, Gemini-pro, LLaMA-70b). The description, data source, data size, and coverage of the dataset used in the experiments are shown in Table 1.

**Table 1:** Dataset statistics for LLMs evaluation experiments

Dataset	Description	Data Source	Data size	Coverage	Presence In KG
Domain Knowledge set	Questions of associations between facial phenotypes, gene, diseases	HPO	100	25 facial phenotypes, 25 genes, 25 diseases	yes
Publication set (selective/non-selective)	Cases from peer-reviewed publications	PubMed publications	200	100 cases with different diseases	yes
GMDB set (selective/non-selective)	Cases from expert-curated database	GMDB^43^	200	100 cases with different diseases	no

The Domain Knowledge set is used in domain knowledge QA.

The Publication set and GMDB set are used in diagnostic tests, temperature analysis, and consistency evaluation.

### Domain knowledge QA

There are complex mechanisms of interaction between facial phenotype, gene, and disease, and we assessed LLMs’ understanding of this area of knowledge by asking about the relationship between the three. Moreover, the knowledge related to facial phenotypes is dispersed across a multitude of literature sources and databases, encompassing vast and diverse information. A considerable number of synonyms and acronyms exist, which can refer to the same concepts.

**Fig. 1:**
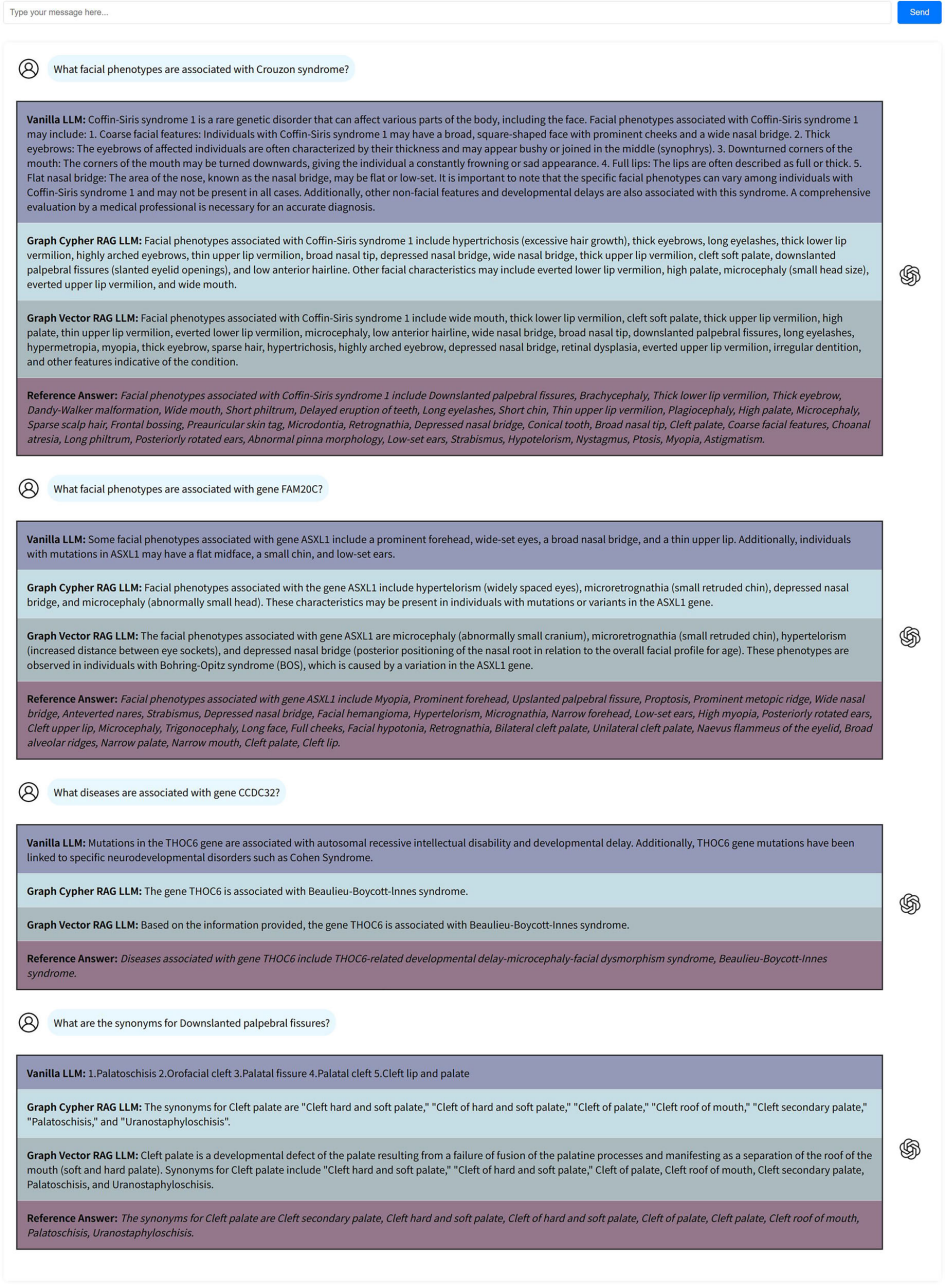
Sample queries on facial phenotypes, genes, and diseases. A set of questions asking about the association between facial phenotype, gene, and disease, as well as synonyms for facial phenotypes. The reference answers are all configured through HPO.

In the domain knowledge QA, the dataset we employed comprises four tasks: (1) facial phenotype-disease association queries, (2) facial phenotype-gene association queries, (3) disease-gene association queries, and (4) facial phenotype synonym queries. Each task consists of 25 questions, each paired with a standardized reference answer formulated by researchers (J.S., B.S.) based on the HPO. To conduct quantitative comparisons, we implemented a dual evaluation approach based on both model-based and rule-based metrics, including BertScore^25^ and coverage. Table 2 presents the quantitative comparison results with reference answers, while Fig. 1 illustrates typical examples of questions for the four tasks.

**Table 2:** Quantitative evaluation results of RAG LLM and Vanilla LLM in Domain Knowledge set

Questions	Methods	BertScore	Coverage
Facial phenotype-disease associations	Vanilla LLM	0.8123	11.52%
	Cypher RAG LLM	0.8621	34.76%
	Vector RAG LLM	**0.8705**	**38.92%**
Facial phenotype-gene associations	Vanilla LLM	0.8127	10.66%
	Cypher RAG LLM	0.8498	30.15%
	Vector RAG LLM	**0.8532**	**40.01%**
Disease-gene associations	Vanilla LLM	0.8377	40.01%
	Cypher RAG LLM	0.8914	75.33%
	Vector RAG LLM	**0.9028**	**80.47%**
Facial phenotype synonyms	Vanilla LLM	0.8743	52.64%
	Cypher RAG LLM	**0.9481**	**88.20%**
	Vector RAG LLM	0.9335	86.50%

For all types of questions, the RAG LLM has a higher BertScore and coverage than the Vanilla LLM. The coverage measures the proportion of key information in reference answers correctly answered by the LLMs. The bold values represent the best performance in this type of question.

The experimental results demonstrate that the vector RAG LLMs outperform in most tasks, and vector matching effectively captures potential fuzzy associations. For instance, the genes ARID1B and ARID1A are treated as entirely distinct entities in Cypher queries but exhibit high similarity in vector space. Additionally, Cypher queries generated by LLMs typically only retrieve direct “Affect” relationships between genes and phenotypes, failing to fully capture multi-hop association paths such as “gene-variant-disease-phenotype.” In comparison, the Cypher RAG LLMs demonstrate unique advantages in structurally straightforward queries due to their precise relationship-matching capability. Notably, both RAG methods deliver superior answers across all questions, whereas the vanilla LLMs occasionally fail to provide valid responses.

Despite their strong performance in other tasks, RAG LLMs achieve relatively low coverage scores (38.92% for phenotype-disease associations and 40.01% for phenotype-gene associations). This discrepancy arises from the inherent asymmetry in these tasks: predicting associations with diseases (fewer targets) is far easier than predicting associations with facial phenotypes (more targets). For example, the EFTUD2 gene is linked to only 2 diseases but 20 distinct facial phenotypes. Since LLMs must cover significantly more facial phenotypes than disease associations, achieving high coverage in phenotype-disease and phenotype-gene tasks is inherently more difficult.

Another limiting factor is the incomplete coverage of KG, as the current FPKG remains insufficient in scope and requires further expansion. A notable advantage of KGs is their dynamic updatability—they can incorporate the latest research findings over time to maintain knowledge currency and accuracy without requiring retraining of LLMs. Despite this limitation, RAG LLMs still demonstrate significant performance improvements over vanilla LLMs across all four tasks, achieving average coverage increases of: Facial phenotype-disease associations ( + 25.32%), Facial phenotype-gene associations ( + 24.42%), Disease-gene associations ( + 37.89%), Facial phenotype synonyms ( + 34.71%).

### Diagnostic tests

The diagnostic test used two datasets. The Publication set and the GMDB set. Each dataset is divided into selective and non-selective subsets. The questions in both subsets were the same, with the selective questions providing alternative answers to help assess the LLMs’ ability to make choices when provided with prompted information. The non-selective questions require the LLMs to generate answers based on its own knowledge and reasoning, which comprehensively evaluates its understanding and reasoning ability. Combining these two types of tests provides a more comprehensive assessment of the LLMs’ performance.

Tables 3, 4 show the performance of two types of RAG LLMs and Vanilla LLMs in Publication set and GMDB set, respectively. The results show that RAG LLMs exhibit significant performance gains in most LLMs, both in selective and non-selective tests. However, Cypher RAG Gemini-pro exhibits a decline in diagnostic accuracy, primarily because Gemini-pro demonstrates minimal capability in generating correct Cypher queries—a specialized query language for graph databases. This limitation prevents it from retrieving relevant information from the KG, thereby reducing diagnostic accuracy.

**Table 3:** Diagnostic accuracy on the publication set

Dataset	Model	Vanilla LLMs	Cypher RAG LLMs	Vector RAG LLMs
Publication set selective test	GPT-3.5-turbo	67.60	**92.40**	90.60
	GPT-4-turbo	90.40	**97.00**	95.00
	GPT-4o	90.20	**95.40**	95.00
	Claude-3-opus	85.20	**94.40**	94.00
	Claude-3-sonnet	63.60	91.20	**92.20**
	Claude-3-haiku	75.60	90.60	**91.00**
	Gemini-pro	54.40	47.60	**92.40**
	LLaMA-70b	72.80	**94.40**	93.20
	Average	74.98	87.88	**92.93**
Publication set non-selective test	GPT-3.5-turbo	49.00	**87.60**	86.20
	GPT-4-turbo	67.40	**95.00**	92.40
	GPT-4o	69.20	**95.00**	93.40
	Claude-3-opus	71.20	**93.40**	92.80
	Claude-3-sonnet	54.00	73.80	**81.80**
	Claude-3-haiku	54.60	71.40	**81.20**
	Gemini-pro	25.20	1.20	**86.00**
	LLaMA-70b	46.20	**88.20**	87.80
	Average	54.60	75.70	**87.70**

Results demonstrate that both Cypher RAG and Vector RAG significantly outperform vanilla LLMs, improving average accuracy by 12.90% and 17.95% in selective tests, and 21.10% and 33.10% in non-selective tests, respectively. The bold values represent the best performance among these three methods.

**Table 4:** Diagnostic accuracy on the GMDB set

Dataset	Model	Vanilla LLMs	Cypher RAG LLMs	Vector RAG LLMs
GMDB set selective test	GPT-3.5-turbo	59.20	65.20	**71.20**
	GPT-4-turbo	85.20	87.00	**88.80**
	GPT-4o	89.20	91.00	**92.80**
	Claude-3-opus	81.20	85.40	**89.60**
	Claude-3-sonnet	63.20	74.80	**86.40**
	Claude-3-haiku	71.20	77.80	**84.20**
	Gemini-pro	51.20	43.00	**66.80**
	LLaMA-70b	70.60	75.40	**80.00**
	Average	71.38	74.95	**82.48**
GMDB set non-selective test	GPT-3.5-turbo	38.40	44.60	**50.80**
	GPT-4-turbo	46.20	57.60	**69.00**
	GPT-4o	48.00	59.80	**71.60**
	Claude-3-opus	52.80	63.80	**74.80**
	Claude-3-sonnet	45.60	58.20	**70.60**
	Claude-3-haiku	46.20	58.00	**69.80**
	Gemini-pro	22.40	0.80	**49.80**
	LLaMA-70b	34.80	43.60	**52.60**
	Average	41.80	48.30	**63.63**

Vector RAG delivers the strongest performance, boosting average accuracy by 11.10% (selective) and 21.83% (non-selective) over vanilla LLMs, with GPT-4o achieving the highest accuracy of 92.80%. Notably, non-selective tests show a steeper performance gap, underscoring RAG’s value in challenging scenarios. The bold values represent the best performance among these three methods.

The results indicate that in non-selective tests, the performance of all LLMs was significantly lower than that in selective tests. Specifically, Vanilla LLMs exhibited average diagnostic accuracy drops of 20.38% and 29.58% on the Publication set and GMDB set, respectively; Cypher RAG LLMs showed relatively smaller declines, yet still reached 12.18% and 26.65%; whereas Vector RAG LLMs displayed the strongest robustness, with diagnostic accuracy reductions limited to 5.23% and 18.85%. Notably, the overall performance of all LLMs (including Vanilla LLMs) on the GMDB set was relatively lower. This may be attributed to GMDB containing a substantial amount of private data that rarely appeared in the training corpora of LLMs.

Although the performance improvements of RAG LLMs on the GMDB set were more moderate compared to those on the Publication set, they still demonstrated significant enhancements. For instance, Vector RAG achieved average diagnostic accuracy improvements of 11.10% and 21.83% in selective/non-selective testing on the GMDB set, while on the Publication set, the improvements were higher at 17.95% and 33.10%. This discrepancy primarily derives from the cases in the GMDB set containing entities and relationships not covered by the KG, thereby limiting the effectiveness of RAG. Unlike the comparable performance of Cypher and Vector RAG on the Publication set, Vector RAG consistently outperformed Cypher RAG on the GMDB set (e.g., achieving +7.53% and +15.33% higher average diagnostic accuracy in selective/non-selective settings, respectively), demonstrating the superior flexibility of vector retrieval: vector embeddings can effectively capture similarities even for out-of-KG entities and better handle partial matches or fuzzy queries, whereas Cypher queries rely on strict schema alignment with the KG.

The primary cause of the performance gap lies in the differences between task requirements and LLMs capabilities. Selective tests benefit from provided answer options, which constrain the solution space and offer implicit guidance; non-selective tests, however, require LLMs to independently generate complete answers through reasoning without structural support. Therefore, we recommend providing as much information as possible about potential diseases or conditions when querying LLMs, as this will significantly enhance the diagnostic accuracy and relevance of responses. Overall, both graph RAG variants consistently improved diagnostic accuracy on the Publication and GMDB sets, and future expansions of the KG are expected to yield further performance enhancements.

### Consistency evaluation

**Fig. 2:**
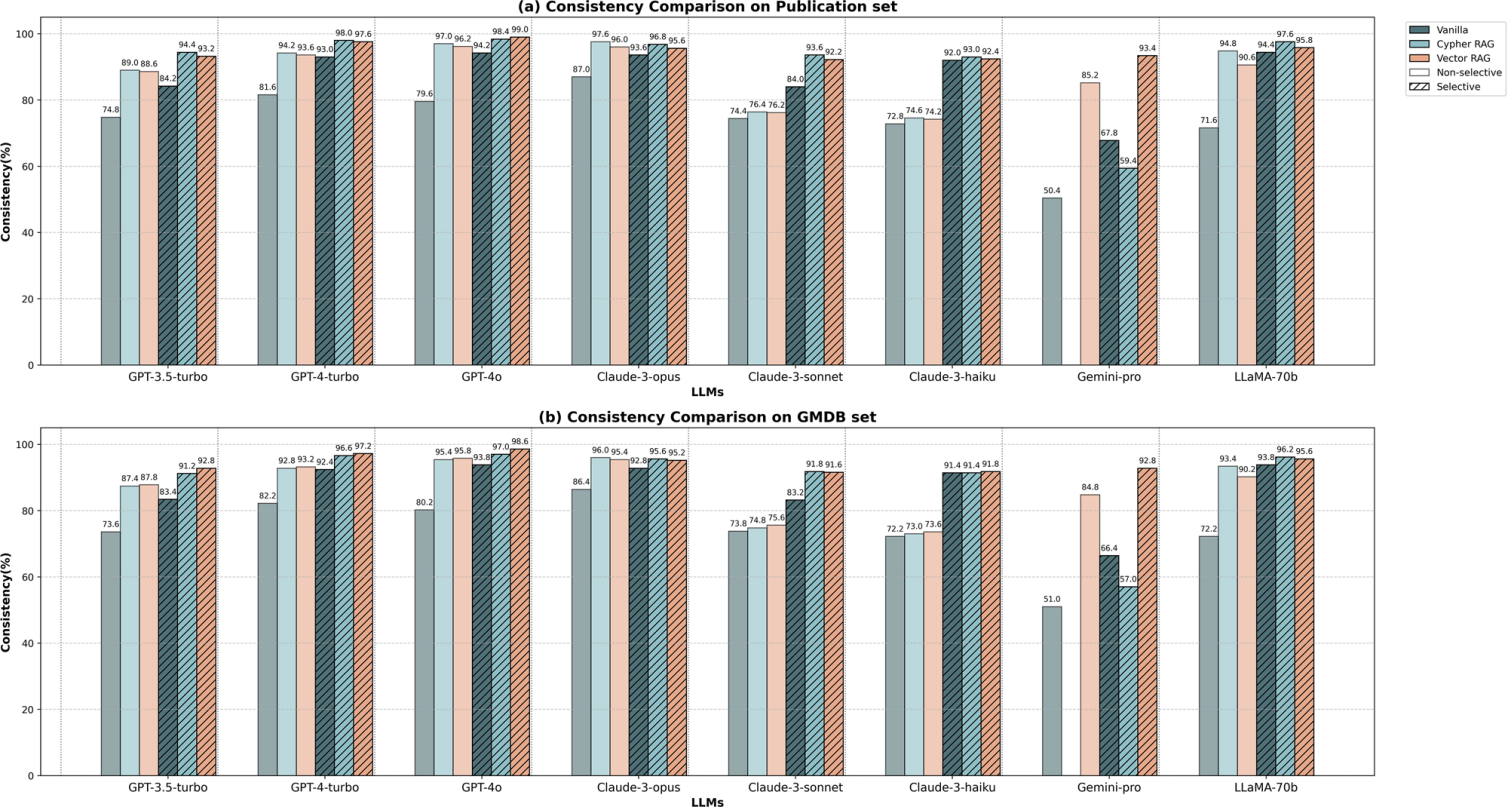
Consistency evaluation. **a** Consistency evaluation on Publication set. Both Cypher RAG and Vector RAG significantly enhance consistency compared to vanilla LLMs, with Vector-RAG-GPT-4o achieving the highest scores (up to 99.0% in selective questions). **b** Consistency evaluation on GMDB set. The overall performance is similar to the publication set, with Vector-RAG-GPT-4o maintaining superior performance (up to 98.6% in selective questions).

LLMs may produce different answers to the same question, therefore self-consistency is a key parameter in evaluating LLMs^26,27^. We asked each LLM the same question 5 times to evaluate the consistency of their answers. Consistency is defined as the proportion of the most frequent answer among five responses to the same question. For example, if three out of five responses were identical, the consistency would be 60%. The consistency evaluation used two datasets. The Publication set and the GMDB set. Each dataset is divided into selective and non-selective subsets. Fig. 2 presents the consistency evaluation results on the Publication set and GMDB set. Both RAG approaches (Cypher and Vector) demonstrated significant and consistent improvements across all datasets. Among all LLMs, GPT-4o exhibited advantages, with Cypher-GPT-4o achieving 99.0% consistency (selective) and 96.2% (non-selective), and Vector-GPT-4o reaching 98.6% (selective) and 95.8% (non-selective).

However, Gemini-pro emerged as an outlier on both datasets. Similar to the Diagnostic Tests, its inability to generate correct Cypher queries led to persistent failure in providing valid answers, rendering consistency metrics uncomputable.

Although RAG LLMs show decreased diagnostic accuracy in the GMDB set, their consistency exhibits similar trends between the Publication set and GMDB set. This phenomenon comes from the deterministic nature of RAG: for identical queries, the system consistently retrieves specific evidence segments, thereby ensuring output reproducibility. It is particularly noteworthy that the system maintains high stability even when the retrieved knowledge contains errors—because this consistency measures the invariance of system responses rather than the correctness of answers.

### Temperature analysis

**Fig. 3:**
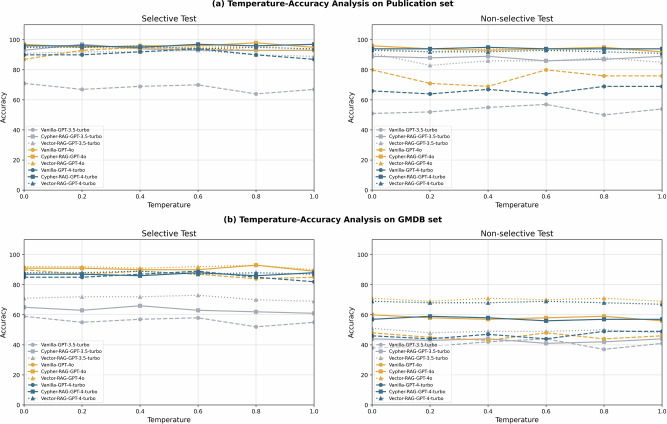
Temperature analysis showing accuracy variations across temperature settings (0.0–1.0) for Vanilla, Cypher-RAG, and Vector-RAG implementations of GPT-3.5-turbo, GPT-4o, and GPT-4-turbo. **a** Temperature analysis of GPTs on Publication set. RAG LLMs are more stable than Vanilla LLMs and tend to produce stable answers with much less effect of temperature. **b** Temperature analysis of GPTs on GMDB set. GMDB set results showing similar trends at reduced accuracy scales.

The temperature parameter of LLMs modulates the degree of stochasticity^28,29^ leading to more diverse outputs and is therefore often referred to as the creativity parameter. Here we evaluated the effect of temperature on the diagnostic accuracy of LLMs, where we use a wide range of temperature *T* = [0,0.2,0.4,0.6,0.8,1.0] for this evaluation. Our results on three representative LLMs, GPT-3.5-turbo, GPT-4o, and GPT-4-turbo are shown in Fig. 3.

To more quantify the performance variability of LLMs under different temperature settings (0.0–1.0), we calculated the standard deviation (σ) of diagnostic accuracy across all temperature folds as shown in Table 5. The RAG σ Reduction metric represents the average reduction in variability achieved by both Cypher-RAG and Vector-RAG implementations compared to Vanilla LLMs. The results indicate that RAG LLMs demonstrated substantially lower σ values (average reduction: 53.94%, range: 28.62–74.29%) compared to their Vanilla counterparts.

**Table 5:** Standard deviation (σ) of diagnostic accuracy across temperature settings (0.0–1.0) for LLMs

Dataset	Model	Vanilla σ	Cypher RAG σ	Vector RAG σ	RAG σ Reduction (%)
Publication set selective test	GPT-3.5-turbo	2.53	1.47	1.60	39.24%
	GPT-4-turbo	2.81	1.17	1.03	60.83%
	GPT-4o	2.35	0.75	0.75	67.90%
Publication set non-selective test	GPT-3.5-turbo	2.64	1.26	2.50	28.62%
	GPT-4-turbo	4.55	1.41	0.98	73.63%
	GPT-4o	2.26	0.41	0.75	74.29%
GMDB set selective test	GPT-3.5-turbo	2.53	1.86	1.47	34.11%
	GPT-4-turbo	2.35	0.89	0.75	64.88%
	GPT-4o	2.28	1.37	1.03	47.40%
GMDB set non-selective test	GPT-3.5-turbo	2.64	1.26	1.17	53.89%
	GPT-4-turbo	2.26	1.03	0.75	60.47%
	GPT-4o	2.07	1.41	0.98	41.97%

Publication set results show Cypher-RAG achieves the lowest variability (σ = 0.41 for GPT-4o in non-selective), while Vector-RAG demonstrates consistent stability (σ ≤ 1.60 in selective). GMDB set exhibits similar patterns. All RAG LLMs show significantly reduced variance compared to Vanilla LLMs (average RAG σ Reduction: 53.94%), suggesting that they perform with less effect of temperature parameter.

The similar trends observed between the Publication and GMDB sets align with our consistency evaluation results. The standard deviation quantifies accuracy fluctuations across different temperature settings, while the consistency metric measures response invariance across repeated queries. Notably, the overall decline in accuracy does not significantly impact these evaluation metrics. Collectively, RAG effectively reduces temperature-induced variability.

## Discussion

In this study, we constructed a facial phenotype domain knowledge graph (FPKG) and combined it with two types of graph RAG (Cypher RAG, Vector RAG) to alleviate the hallucination of LLMs and improve their ability to answer questions in specialized domains. To systematically evaluate the effectiveness of this approach, we developed three benchmark datasets comprising 500 rare genetic disease-related questions, quantitatively comparing the performance differences between RAG LLMs and vanilla LLMs. Specifically, we conducted four specialized experiments across eight distinct LLMs: (1) domain-specific knowledge question answering, (2) diagnostic testing, (3) consistency evaluation, and (4) temperature analysis. Results demonstrate that graph RAG techniques significantly improve the performance of LLMs in the field of rare genetic diseases.

In domain knowledge QA, we conducted a qualitative analysis. The experimental results demonstrate that the vector RAG LLM outperformed in most tasks, as vector matching effectively captured latent ambiguous associations. For instance, the genes ARID1B and ARID1A were treated as entirely distinct entities in Cypher queries but exhibited high similarity in the vector space. Notably, both RAG methods significantly improved the performance of vanilla LLMs.

In diagnostic tests, RAG LLMs demonstrate significant accuracy improvements across most LLMs. Specifically, GPT-4-turbo, GPT-4o, and Claude-3-opus showed extremely high levels of diagnostic accuracy. Although the performance enhancement of RAG LLMs on the GMDB set was more modest compared to that on the Publication set, they still displayed significant improvements. This is because RAG LLMs retained diagnostic utility through negative evidence-driven reasoning. For instance, a patient’s disease was entirely absent from the FPKG. However, it retained the ability to differentiate between CHARGE syndrome and 22q11.2 deletion syndrome^30^—conditions with overlapping clinical presentations but distinct pathogenic mechanisms—even if the latter was unrecorded in FPKG. Retrieved knowledge enabled the exclusion of genetically distinct CHARGE syndrome (associated with CHD7 mutations). This exclusion process effectively narrowed the diagnostic hypothesis space, allowing LLMs to converge toward the correct diagnosis through elimination. The results show that the RAG approaches can significantly improve the performance of the LLMs in complex diagnostic tasks.

Li et al.^31^ evaluated the consistency of GPTs under different prompt templates, and here in the consistency evaluation, we studied and analyzed the consistency of RAG LLMs and extended the study to examine LLMs such as Claude, LLaMA, etc., and the results showed that the consistency of RAG LLMs was higher than that of the Vanilla LLMs, with GPT-4-turbo and Claude-3-opus exhibiting the highest consistency.

In a previous study, Fares et al.^32^ found that LLMs with a temperature setting of 0.3 performed better in answering ophthalmology-related questions, in our study we analyzed the sensitivity of the LLMs to temperature, and the results showed that RAG effectively reduces temperature-induced variability, and tend to produce stable answers.

This study has several strengths:

- **Rare genetic disease datasets:** We constructed three datasets comprising a total of 500 questions to provide standardized benchmarks for the quantitative evaluation of LLMs.
- **Standardized terminology:** medical knowledge comes from different literatures and databases with great heterogeneity, and our KG is constructed based on human phenotype ontology, which makes the responses of LLMs more terminologically standardized.
- **Dynamic knowledge**: the KG is not static and can be updated over time to incorporate the latest research and keep the knowledge up-to-date and accurate.

This study also has some limitations. First, each of these two approaches has its own limitations, Cypher RAG performs worse than the Vanilla version when combined with LLMs that have weak cypher generation capabilities, such as Gemini-pro. Vector RAG, on the other hand, is not limited by its ability to generate cypher queries, but is not able to query the KG as accurately as Cypher RAG.

Second, RAG LLMs rely heavily on the data of the KG, while our KG does not fully cover the knowledge of this domain, and when no relevant information can be queried in the graph, the LLMs resort to their pretrained knowledge^33–35^, which may generate hallucinations despite offering broader coverage. The size and scope of the KG can be further expanded in future studies to cover more facial phenotypes and related genetic diseases. In addition, more advanced retrieval mechanisms can be explored to improve the accuracy and efficiency of retrieval queries.

Future studies can further explore the application of RAG LLMs in the following areas: (1) From the physician’s perspective, RAG LLMs can assist physicians in quickly recognizing diseases, especially when dealing with complex rare cases, RAG LLMs can help by answering facial phenotypic features associated with specific rare genetic diseases, and can be used as a teaching tool to enhance medical students’ and physicians’ understanding of the facial features of rare genetic diseases. (2) From the patient’s perspective, RAG LLMs can provide more standardized responses in specific domains to help patients and families better understand the disease and improve the convenience of access to medical information. (3) From the researcher’s perspective, RAG LLMs can assist researchers in deeply analyzing the complex facial phenotype-gene-disease relationships and uncover more potential associations and patterns.

This study investigates how LLMs and KGs can address the unique diagnostic barriers in rare genetic diseases—conditions where limited clinical data, phenotypic heterogeneity, and low physician familiarity often delay accurate identification^36,37^. By integrating LLMs with domain-specific KGs (which provide structured, curated knowledge), this approach reduces hallucinations in LLMs, thereby enhancing their reliability and accuracy. The method is particularly critical for low-prevalence, high-stakes conditions, where even marginal gains in accuracy can significantly improve patient outcomes. Evaluation results demonstrate that RAG LLMs not only improve diagnostic accuracy across all tested models but also exhibit greater consistency and effectively mitigate temperature-induced variability. Among the evaluated LLMs in the rare genetic disease domain, GPT-4-turbo, GPT-4o, and Claude-3-Opus demonstrated the strongest performance.

## Methods

### Knowledge graph construction

**Fig. 4:**
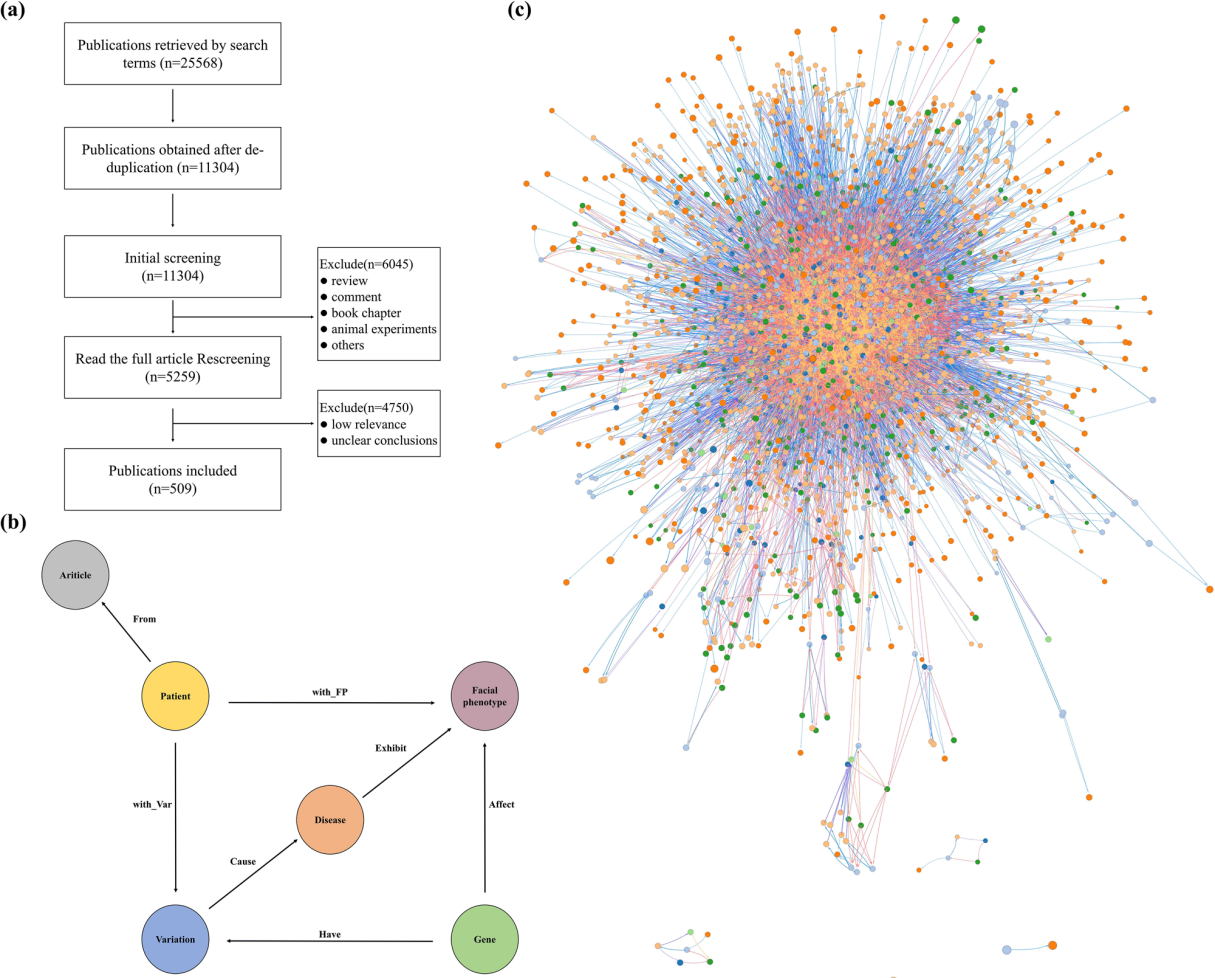
The process of knowledge graph construction. **a** The screening process. A total of 25,568 relevant publications were retrieved, and 509 publications remained after screening. **b** The structure of FPKG. The entities were categorized into five types, i.e., Facial phenotype, Gene, Disease, Patient, and Article. The relationships of these five entities were categorized into seven: Affect, Cause, From, Exhibit, Have, with_FP, with_Var. **c** Overview of FPKG using GraphXR. The knowledge graph includes 6,143 nodes and 19,282 relationships.

First, our research conducted a comprehensive search of the Pubmed database using search terms generated by HPO, and 509 relevant publications on facial phenotype-associated rare genetic diseases were selected after screening, the screening process is shown in Fig. 4a.

Second, Fig. 4b shows the structure of the FPKG. Based on this structure, we used tools such as PhenoTagger^38^, PubTator^39^, and Esearch^40^ to extract the entities, and then manually identified the relationships between them. After several manual reviews, the structured data was formatted and stored in the Neo4j graphical database.

Finally, Fig. 4**c** shows the visualization of the complete KG, which makes the understanding of content and relationships more intuitive.

**Fig. 5:**
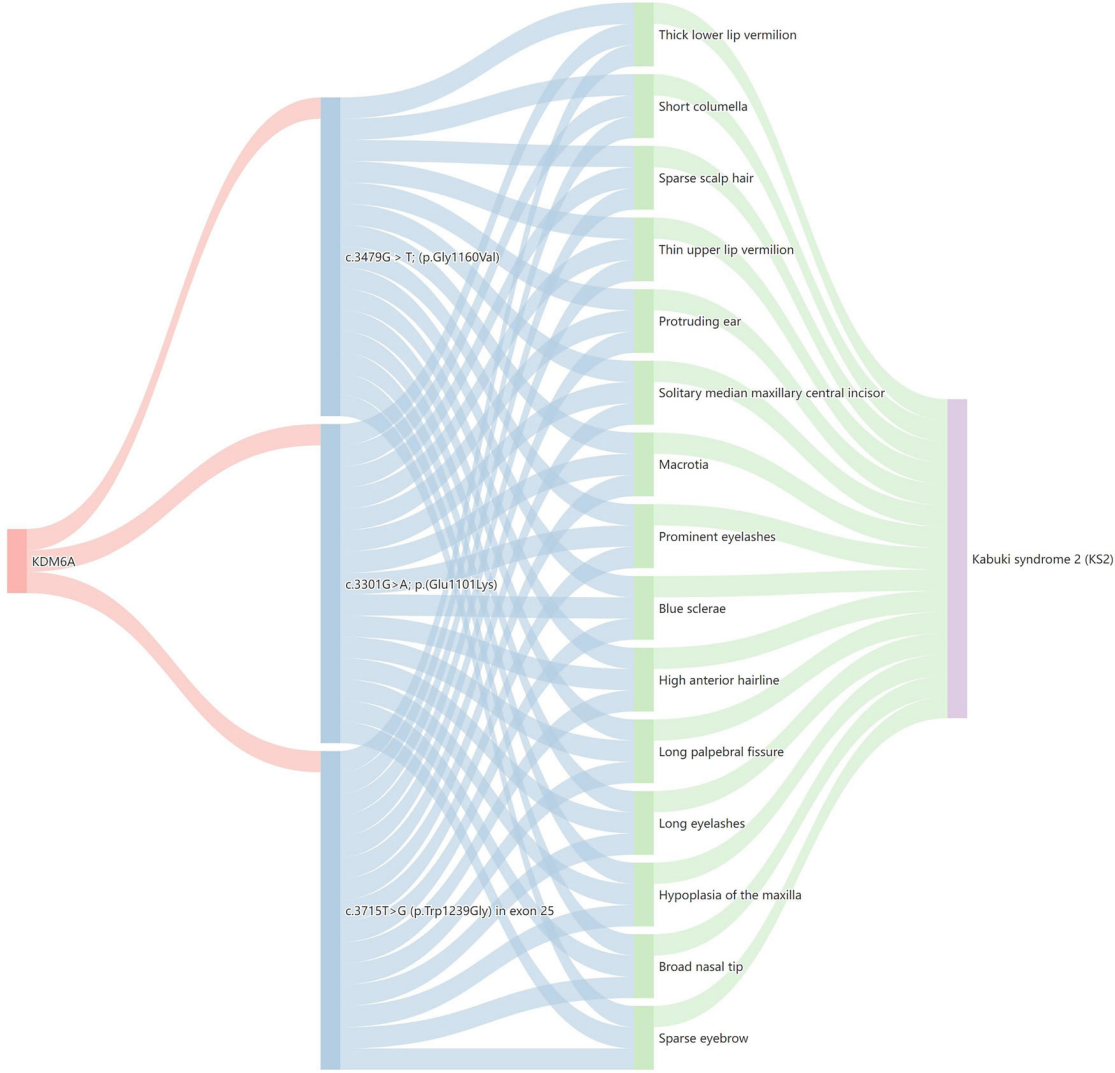
Sankey diagram analysis of KDM6A. The diagram is structured into four layers, starting with the KDM6A gene in the first layer, followed by variant details in the second layer. The third layer displays associated facial phenotypes, and the fourth layer details rare genetic diseases linked to these variants.

Table 6 shows the statistical information of FPKG, including six types of nodes and seven relationships. Fig. 5 shows the Sankey diagram analysis of KDM6A(URL), more results of the Sankey diagram analysis are shown in the Supplementary Figs. 1–4.

**Table 6:** Knowledge graph statistics, including 6 node types and 7 relationship types

Type	Label	Count	Description
Node	Article	509	Publication node
	Disease	225	Disease node
	Genotype	397	Genotype node
	Variation	1242	Gene Variation node
	FacePhenotype	2623	Facial Phenotype node
	Sample	1147	Patient node
Relation	Affect	4296	Relationship between genotype and facial phenotype
	Cause	748	Relationship between gene variation and disease
	Exist	1147	Relationship between sample and article
	Has_Phenotype	2598	Relationship between disease and facial phenotype
	Have	1242	Relationship between genetic variation and genotype
	Mention_FP	8012	Relationship between sample and facial phenotype
	Mention_Var	1239	Relationship between genetic variation and disease

### Retrieval-augmented generation

Graph Cypher Retriever: Upon receiving a user query, cypher queries are generated through an LLM to find relevant information in the KG. This queried knowledge is then entered into the LLM as contextual prompts along with user prompts to generate the final response. This approach utilizes the structured data in the KG to ensure the accuracy and relevance of the answer.

Graph Vector Retriever: Upon receiving a user query, firstly the well-trained Named Entity Recognition (NER) model identifies the entities in the query, which is then added as a node to the KG and the graph is embedded as vector representation, and finally the K nodes with the highest cosine similarity and their neighbors as a subgraph to construct context prompts, which are inputted into the LLM along with the user prompts to generate the final response.

**Fig. 6:**
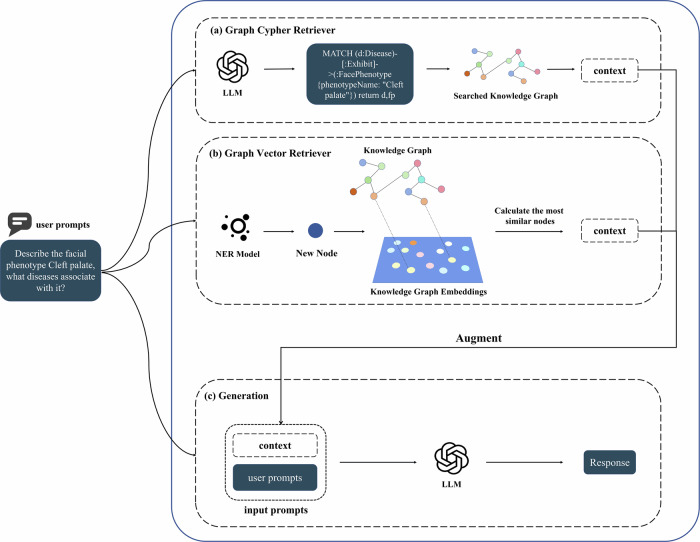
The pipeline of two types of Graph RAG. **a** Graph Cypher Retriever. A LLM generates cypher queries to retrieve relevant information from the KG as context. **b** Graph Vector Retriever. A well-trained NER model identifies entities in the query as nodes to be added to the KG and embedded as vector representations, and finally, the K nodes with the highest cosine similarity and their neighbors are used as subgraphs to build the context. **c** Generation. The retrieved context is entered into LLM along with the user prompt to get the final response.

In particular, Graph Cypher Retriever is able to accurately locate the most suitable context in the KG but its effectiveness depends on the ability of the LLM to generate cypher queries. Graph Vector Retriever, on the other hand, is able to identify and retrieve the most similar subgraph to the query as contexts, which makes it effective even when cypher generation is weak. Graph cypher retriever and graph vector retriever pipelines are shown in Fig. 6.

NER Model: Named Entity Recognition (NER) has been widely used in the biomedical field. For example, BioBERT^41^ is capable of recognizing specific biomedical entities, while PhenoTagger^38^ excels in recognizing HPO concepts. However, there is no NER model that can comprehensively cover all the key information in this field, such as basic patient information (age, gender, race), genetic variation information (genes, mutations), and phenotypic information (facial phenotypes).

In view of this, we curated a comprehensive NER corpus, which contains information on the three types mentioned above. Considering the difficulty of reproducing the existing NER models, we chose to train on Bert-base. The NER corpus and model weights were published in our Github and huggingface, respectively, and subsequently applied to the graph vector retriever. The validation metrics of our NER model are shown in Supplementary Table 3.

Graph Embedding Algorithm: In graph vector retriever, we need to transform the KG into vector representation while ensuring that as much key information as possible is retained. To evaluate which graph embedding algorithms are able to preserve the most graph structure and feature information, we implemented a node classification task on FPKG, firstly, different graph embedding algorithms were used to transform the graph into vector representation with the types of nodes as labels, and random forest was used to predict the labels of the test set, to evaluate the F1 score of different algorithms.

In this way, we can quantify the efficacy of various embedding algorithms in preserving graph structure and feature information, and then select the optimal graph embedding algorithm to improve the overall retrieval accuracy. We evaluate several graph embedding algorithms as shown in Table 7, and GLEE^42^ performs the best.

**Table 7:** Graph embedding algorithms evaluation

Model	Micro-F1	Macro-F1	Weighted-F1
DeepWalk^45^	0.59	0.29	0.50
Node2Vec^46^	0.60	0.30	0.51
Walklets^47^	0.75	0.43	0.70
SocioDim^48^	0.77	0.51	0.76
RandNE^49^	0.53	0.24	0.44
NetMF^50^	0.74	0.49	0.73
**GLEE**^42^	**0.78**	**0.54**	**0.77**

The goal is to select algorithms that maximize the preservation of our knowledge graph structure and feature information, GLEE performs the best.

### Datasets construction

To quantitatively evaluate LLMs’ performance in the domain of rare genetic diseases, we designed three benchmark datasets. The description, data source, data size, and coverage of the dataset used in the experiments are shown in Table 1.

The Domain knowledge set comprises four tasks: (1) facial phenotype-disease association queries, (2) facial phenotype-gene association queries, (3) disease-gene association queries, and (4) facial phenotype synonym queries. Each task consists of 25 questions, each paired with a standardized reference answer formulated by researchers (J.S., B.S.) based on the HPO.

The Publication set and GMDB set comprises diagnostic test questions that include patient demographics (age, gender, ethnicity), genetic mutations, and facial phenotypes, requiring LLMs to generate precise diagnoses. Both sets contain two subsets featuring identical questions but presented in distinct formats (selective and non-selective), each consisting of 100 questions. Selective questions provide LLMs with predefined answer options, thereby facilitating the evaluation of their capacity to make informed selections based on the provided information. Conversely, non-selective questions require LLMs to autonomously generate responses by leveraging their intrinsic knowledge and reasoning capabilities, offering a more comprehensive assessment. When employed in combination, these two subsets provide a more thorough evaluation of the LLMs’ performance.

The Publication set consists of cases collected from the published research literature, while the GMDB set is sourced from the GestaltMatcher Database^43^ (GMDB), an expert-curated rare genetic disease database. In the selective questions, each question presented four options: one correct answer paired with three incorrect options randomly selected from a pool of 225 diseases (encompassing the full spectrum of diseases in FPKG). For the GMDB set, the incorrect options were randomly chosen from a larger pool of 581 diseases (encompassing the full spectrum of diseases in GMDB).

The three datasets are all publicly available on our GitHub repository (https://github.com/zhelishisongjie/Facial-Phenotype-RAG). An example of a diagnostic question is shown in Table 8, with patients from this research^44^.

**Table 8:** An example of diagnostic question

Selective question	Non-selective question	Answer	Source
There is a 5.0-year-old female Turkish patient with a mutation in CHD7, namely, c. 2443-2 A > G and with facial phenotypes of Wide nasal bridge, Posteriorly rotated ears, Thin upper lip vermilion, Chorioretinal coloboma, Protruding ear, Proptosis. What disease might this patient have? Choose from the following options: A.Floating-Harbor syndrome (FLHS) B.CHARGE syndrome C.Noonan syndrome-like disorder with loose anagen hair 2 (NSLH2) D.Tricho-rhino-phalangeal syndrome,TYPE I(TRPS1)	There is a 5.0-year-old female Turkish patient with a mutation in CHD7, namely, c. 2443-2 A > G and with facial phenotypes of Wide nasal bridge, Posteriorly rotated ears, Thin upper lip vermilion, Chorioretinal coloboma, Protruding ear, Proptosis. What disease might this patient have?	CHARGE syndrome	10.1016/j.gene.2015.11.006

### Implementation details

In this study, we made the relevant code, KG, corpus, and NER model publicly available with the aim of increasing the transparency of the study and providing the necessary detailed information, and these resources are available in our GitHub repository (https://github.com/zhelishisongjie/Facial-Phenotype-RAG).

The prompt templates used for Cypher generation, Vanilla LLMs, and RAG LLMs are provided in Supplementary Table 1. The temperature parameter was set to 0.1 for the domain knowledge QA, diagnostic tests, and consistency evaluation. The LLMs were all queried via API, and the introduction to LLMs is shown in Supplementary Table 2.

NER training: We train the model for 100 epochs with a batch size of 32 using the Adam optimizer at a learning rate of 5e-5, employing early stopping based on validation F1 score as our primary metric. Validation metrics include precision, recall, and F1-score. The validation performance of our NER model is shown in Supplementary Table 3.

Domain knowledge QA: The questions were designed to cover associations between facial phenotypes, genes, and diseases. The terms were selected from the HPO and manually constructed into questions. The reference answer formulated by researchers (J.S., B.S.) based on the HPO. The evaluation metrics include BertScore^25^ and coverage, where BertScore assesses the semantic similarity between the LLM answer and the reference answer, and coverage assesses how many of the points made in the LLM answer can be found in the reference answer, ensuring that the answer is not only relevant but also accurate. For example, the reference answer contains **23** phenotypes and the Vanilla LLM mentions **4** of them, resulting in a calculated coverage of **17.39%**. In this section, the LLM we assessed is GPT-3.5-turbo.

Diagnostic Tests: For both the Publication set and GMDB set, we conducted five repeated queries for each question across all LLMs. Each response was manually evaluated for correctness. The final performance metric was derived by calculating the average accuracy across all five queries.

Consistency Evaluation: Based on the diagnostic test responses, we assessed the consistency of answers by querying each question five times and calculating the average consistency score. Consistency was defined as the proportion of the most frequent answer among the five responses to a given question. For example, if three out of five responses were identical, the consistency would be 60%. In this evaluation, only the specific disease identified by the LLM was considered; any additional explanatory content was excluded from the consistency analysis.

Temperature Analysis: We systematically evaluated temperature effects across a broad range (T = [0.0, 0.2, 0.4, 0.6, 0.8, 1.0]), conducting single queries per temperature point for each question. Diagnostic accuracy was then calculated for each LLM under different temperature conditions. In this experiment, we select GPT-3.5-turbo, GPT-4o, GPT-4-turbo for analysis. The RAG σ Reduction metric represents the average reduction in variability achieved by both Cypher-RAG and Vector-RAG implementations compared to Vanilla LLMs.

## Supplementary information

Supplementary Information

## Data Availability

The datasets used in this study are available in our GitHub repository: https://github.com/zhelishisongjie/Facial-Phenotype-RAG. Under the GMDB's restricted access agreement, we have masked all sensitive information as ***** but retained patient IDs. To reproduce our results, you may apply for GMDB access (https://db.gestaltmatcher.org/) and use these patient IDs.

## Code availability

The code and its description, required packages, installation tutorials, specific parameters are publicly available in our GitHub repository: https://github.com/zhelishisongjie/Facial-Phenotype-RAG.

## References

[1] Li, Z.-L. et al. FGFR2 mutation in a Chinese family with unusual Crouzon syndrome. *Int. J. Ophthalmol.* **9**, 1403 (2016). 27803855 10.18240/ijo.2016.10.06 PMC5075653

[2] Gurovich, Y. et al. Identifying facial phenotypes of genetic disorders using deep learning. *Nat. Med.* **25**, 60–64 (2019). 30617323 10.1038/s41591-018-0279-0

[3] Hsieh, T.-C. et al. GestaltMatcher facilitates rare disease matching using facial phenotype descriptors. *Nat. Genet.* **54**, 349–357 (2022). 35145301 10.1038/s41588-021-01010-x PMC9272356

[4] Dingemans, A. J. et al. PhenoScore quantifies phenotypic variation for rare genetic diseases by combining facial analysis with other clinical features using a machine-learning framework. *Nat. Genet.* **55**, 1598–1607 (2023). 37550531 10.1038/s41588-023-01469-w PMC11414844

[5] Vaswani, A. et al. Attention is all you need. *Advances in neural information processing systems* **30** (2017).

[6] Achiam, J. et al. Gpt-4 technical report. *arXiv preprint**arXiv:2303.08774* (2023).

[7] Anthropic. *Introducing the next generation of Claude*, <https://www.anthropic.com/news/claude-3-family> (2024).

[8] Team, G. et al. Gemini: A family of highly capable multimodal models. *arXiv preprint**arXiv:2312.11805* (2023).

[9] Touvron, H. et al. Llama 2: Open foundation and fine-tuned chat models. *arXiv preprint**arXiv:2307.09288* (2023).

[10] Savage, T., Nayak, A., Gallo, R., Rangan, E. & Chen, J. H. Diagnostic reasoning prompts reveal the potential for large language model interpretability in medicine. *NPJ Digital Med.* **7**, 20 (2024). 10.1038/s41746-024-01010-1 PMC10808088 38267608

[11] Spiegel, B. M. et al. Feasibility of combining spatial computing and AI for mental health support in anxiety and depression. *NPJ Digital Med.* **7**, 22 (2024). 10.1038/s41746-024-01011-0 PMC10817913 38279034

[12] Luu, R. K. & Buehler, M. J. BioinspiredLLM: Conversational Large Language Model for the Mechanics of Biological and Bio-Inspired Materials. *Adv. Sci.* **11**, 2306724 (2024). 10.1002/advs.202306724 PMC10933662 38145334

[13] Wang, H., Gao, C., Dantona, C., Hull, B. & Sun, J. DRG-LLaMA: Tuning LLaMA model to predict diagnosis-related group for hospitalized patients. *npj Digital Med.* **7**, 16 (2024). 10.1038/s41746-023-00989-3 PMC10803802 38253711

[14] Waisberg, E., Ong, J., Masalkhi, M. & Lee, A. G. Large language model (LLM)-driven chatbots for neuro-ophthalmic medical education. *Eye* **38**, 639–641 (2024). 37749374 10.1038/s41433-023-02759-7 PMC10920622

[15] Ji, Z. et al. Survey of hallucination in natural language generation. *ACM Comput. Surv.* **55**, 1–38 (2023).

[16] Kandpal, N., Deng, H., Roberts, A., Wallace, E. & Raffel, C. In *International Conference on Machine Learning*. 15696-15707 (PMLR).

[17] Chen, J., Lin, H., Han, X. & Sun, L. In *Proceedings of the AAAI Conference on Artificial Intelligence*. 17754-17762.

[18] Gao, Y. et al. Retrieval-augmented generation for large language models: A survey. *arXiv preprint**arXiv:2312.10997* (2023).

[19] Sanmartin, D. K. G.-R. A. G.: Bridging the Gap Between Knowledge and Creativity. *arXiv preprint**arXiv:2405.12035* (2024).

[20] Jiang, X. et al. Think and retrieval: A hypothesis knowledge graph enhanced medical large language models. *arXiv preprint**arXiv:2312.15883* (2023).

[21] Cavalleri, E. et al. SPIREX: Improving LLM-based relation extraction from RNA-focused scientific literature using graph machine learning. *Proceedings of the VLDB Endowment*. *ISSN* **2150**, 8097

[22] Buehler, M. J. Accelerating scientific discovery with generative knowledge extraction, graph-based representation, and multimodal intelligent graph reasoning. *Mach. Learn. Sci. Technol.* **5**, 035083 (2024).

[23] Buehler, M. J. Generative retrieval-augmented ontologic graph and multiagent strategies for interpretive large language model-based materials design. *ACS Eng. Au* **4**, 241–277 (2024). 38646516 10.1021/acsengineeringau.3c00058 PMC11027160

[24] Köhler, S. et al. The human phenotype ontology in 2021. *Nucleic acids Res.* **49**, D1207–D1217 (2021). 33264411 10.1093/nar/gkaa1043 PMC7778952

[25] Zhang, T., Kishore, V., Wu, F., Weinberger, K. Q. & Artzi, Y. Bertscore: Evaluating text generation with bert. *arXiv preprint**arXiv:1904.09675* (2019).

[26] Wang, X. et al. Self-consistency improves chain of thought reasoning in language models. *arXiv preprint**arXiv:2203.11171* (2022).

[27] Omiye, J. A., Lester, J. C., Spichak, S., Rotemberg, V. & Daneshjou, R. Large language models propagate race-based medicine. *NPJ Digital Med.* **6**, 195 (2023). 10.1038/s41746-023-00939-z PMC10589311 37864012

[28] Peeperkorn, M., Kouwenhoven, T., Brown, D. & Jordanous, A. Is temperature the creativity parameter of large language models? *arXiv preprint**arXiv:2405.00492* (2024).

[29] Zhu, Y. et al. In *Proceedings of the AAAI Conference on Artificial Intelligence*. 437-445. PMC8159182 34055465

[30] Corsten-Janssen, N. et al. More clinical overlap between 22q11. 2 deletion syndrome and CHARGE syndrome than often anticipated. *Mol. Syndromol.* **4**, 235–245 (2013). 23885230 10.1159/000351127 PMC3711480

[31] Wang, L. et al. Prompt engineering in consistency and reliability with the evidence-based guideline for LLMs. *npj Digital Med.* **7**, 41 (2024). 10.1038/s41746-024-01029-4 PMC10879172 38378899

[32] Antaki, F. et al. Capabilities of GPT-4 in ophthalmology: An analysis of model entropy and progress towards human-level medical question answering. *British Journal of Ophthalmology* (2023). 10.1136/bjo-2023-324438 37923374

[33] Ke, Y. H. et al. Retrieval augmented generation for 10 large language models and its generalizability in assessing medical fitness. *npj Digital Med.* **8**, 187 (2025). 10.1038/s41746-025-01519-z PMC11971376 40185842

[34] Ge, J. et al. Development of a liver disease–specific large language model chat interface using retrieval-augmented generation. *Hepatology* **80**, 1158–1168 (2024). 38451962 10.1097/HEP.0000000000000834 PMC11706764

[35] Wang, D. et al. Enhancement of the performance of large language models in diabetes education through retrieval-augmented generation: Comparative study. *J. Med. Internet Res.* **26**, e58041 (2024). 39046096 10.2196/58041 PMC11584532

[36] Ferreira, C. R. The burden of rare diseases. *Am. J. Med. Genet. Part A* **179**, 885–892 (2019). 30883013 10.1002/ajmg.a.61124

[37] Baird, P. A., Anderson, T. W., Newcombe, H. B. & Lowry, R. Genetic disorders in children and young adults: a population study. *Am. J. Hum. Genet.* **42**, 677 (1988). 3358420 PMC1715177

[38] Luo, L. et al. PhenoTagger: A hybrid method for phenotype concept recognition using human phenotype ontology. *Bioinformatics* **37**, 1884–1890 (2021). 33471061 10.1093/bioinformatics/btab019 PMC11025364

[39] Wei, C.-H., Kao, H.-Y. & Lu, Z. PubTator: a web-based text mining tool for assisting biocuration. *Nucleic acids Res.* **41**, W518–W522 (2013). 23703206 10.1093/nar/gkt441 PMC3692066

[40] Buchmann, J. P. & Holmes, E. C. Entrezpy: A Python library to dynamically interact with the NCBI Entrez databases. *Bioinformatics* **35**, 4511–4514 (2019). 31077305 10.1093/bioinformatics/btz385 PMC6821292

[41] Lee, J. et al. BioBERT: A pre-trained biomedical language representation model for biomedical text mining. *Bioinformatics* **36**, 1234–1240 (2020). 31501885 10.1093/bioinformatics/btz682 PMC7703786

[42] Torres, L., Chan, K. S. & Eliassi-Rad, T. GLEE: Geometric Laplacian eigenmap embedding. *J. Complex Netw.* **8**, cnaa007 (2020).

[43] Lesmann, H. et al. GestaltMatcher Database-A global reference for facial phenotypic variability in rare human diseases. *Res. Square*, rs. 3. rs-4438861 (2024).

[44] Lee, B. et al. Revealing the function of a novel splice-site mutation of CHD7 in CHARGE syndrome. *Gene* **576**, 776–781 (2016). 26551301 10.1016/j.gene.2015.11.006

[45] Perozzi, B., Al-Rfou, R. & Skiena, S. In *Proceedings of the 20th ACM SIGKDD international conference on Knowledge discovery and data mining*. 701-710.

[46] Grover, A. & Leskovec, J. In *Proceedings of the 22nd ACM SIGKDD international conference on Knowledge discovery and data mining*. 855-864. 10.1145/2939672.2939754 PMC5108654 27853626

[47] Perozzi, B., Kulkarni, V., Chen, H. & Skiena, S. In *Proceedings of the 2017 IEEE/ACM International Conference on Advances in Social Networks Analysis and Mining**2017*. 258-265.

[48] Tang, L. & Liu, H. In *Proceedings of the 15th ACM SIGKDD international conference on Knowledge discovery and data mining*. 817-826.

[49] Zhang, Z., Cui, P., Li, H., Wang, X. & Zhu, W. In *2018**IEEE international conference on data mining (ICDM)*. 787-796 (IEEE).

[50] Qiu, J. et al. In *Proceedings of the eleventh ACM international conference on web search and data mining*. 459-467.

