# Publisher Correction: Graph retrieval augmented large language models for facial phenotype associated rare genetic disease

**DOI:** 10.1038/s41746-025-02017-y

**Published:** 2025-10-08

**Authors:** Jie Song, Zhichuan Xu, Mengqiao He, Jinhua Feng, Bairong Shen

**Affiliations:** 1 Department of Ophthalmology and Institutes for Systems Genetics, Frontiers Science Center for Disease-related Molecular Network, West China Hospital, Sichuan University, Chengdu, China; 2 School of Computer Science and Software Engineering, Southwest Petroleum University, Chengdu, China

**Keywords:** Diagnosis, Literature mining, Machine learning

Correction to: *npj Digital Medicine* 10.1038/s41746-025-01955-x, published online 24 August 2025

In the originally accepted manuscript, Dr. Bairong Shen was specified as the sole corresponding author. However, in the published version, all authors were incorrectly marked as corresponding authors.

