# Supplementary material for: Graph retrieval augmented large language models for facial phenotype associated rare genetic disease
Source: NPJ Digit Med. 2025 Aug 24;8:543. doi: 10.1038/s41746-025-01955-x (PMC12375027; doi:10.1038/s41746-025-01955-x)
Supplement: Supplementary file 1 — Supplementary Information [file 41746_2025_1955_MOESM1_ESM.pdf]

SUPPLEMENTARY INFORMATION

Supplementary Table 1. Prompt Templates for Different Query Types

Query Type	Prompt Template
Cypher Generation	CYPHER_GENERATION_TEMPLATE = """<br>You are a Neo4j Cypher query expert. Please generate Cypher statements to query a graph database based on user questions.<br><br>Instructions:<br>Your generated Cypher query must follow Neo4j Graph database Schema provided.<br>Do not include any explanations or apologies in your responses.<br>Do not respond to any questions that might ask anything else than for you to construct a Cypher statement.<br>Do not include any text except the generated Cypher statement.<br><br>Schema: [schema]<br>The question is: [question]<br>"""
Vanilla LLM Prompt	Vanilla_QA_TEMPLATE="""<br>You are a medical genetics assistant specializing in analyzing facial phenotypes to identify rare genetic diseases, and interpret the relationships between genes, facial features, and associated diseases.<br><br>The question is: [question]<br>"""
RAG LLM Prompt	QA_TEMPLATE="""<br>You are a medical genetics assistant specializing in analyzing facial phenotypes to identify rare genetic diseases, and interpret the relationships between genes, facial features, and associated diseases.<br><br>Instructions:<br>The information section provides some knowledge based on the patient's symptoms and genetic data. You should refer to this knowledge and make the most likely diagnosis.<br>Make the answer sound as a response to the question.<br>Do not mention that you got this result based on the information provided, but ensure the explanation is medically sound and justifiable.<br>If the information provided is empty, answer the question normally using medical reasoning based on typical symptoms and known genetic associations.<br><br>Information: [context]<br>The question is: [question]<br>"""

Supplementary Table 2. Details of the large language models used.

LLM	LLM full name	Context window (tokens)
GPT-3.5-turbo	gpt-3.5-turbo-0125	16,358
GPT-4-turbo	gpt-4-turbo-2024-04-09	128,000
GPT-4o	gpt-4o-2024-05-13	128,000
Claude-3-opus	claude-3-opus-20240229	200,000
Claude-3-sonnet	claude-3-sonnet-20240229	200,000
Claude-3-haiku	claude-3-haiku-20240307	200,000
Gemini-pro	gemini-pro	32,768
LLaMA-70b	llama3-70b-8192	8,192

Supplementary Table 3. Named entity recognition model validation results showing micro-average, macro-average and weighted-average performance metrics.

Model	Average	Precision	Recall	F1-score
NER model	Micro average	0.97	0.98	0.97
	Macro average	0.93	0.95	0.94
	Weighted average	0.97	0.98	0.97

Supplementary Figure 1 shows the Sankey diagram analysis of ASXL3(URL),

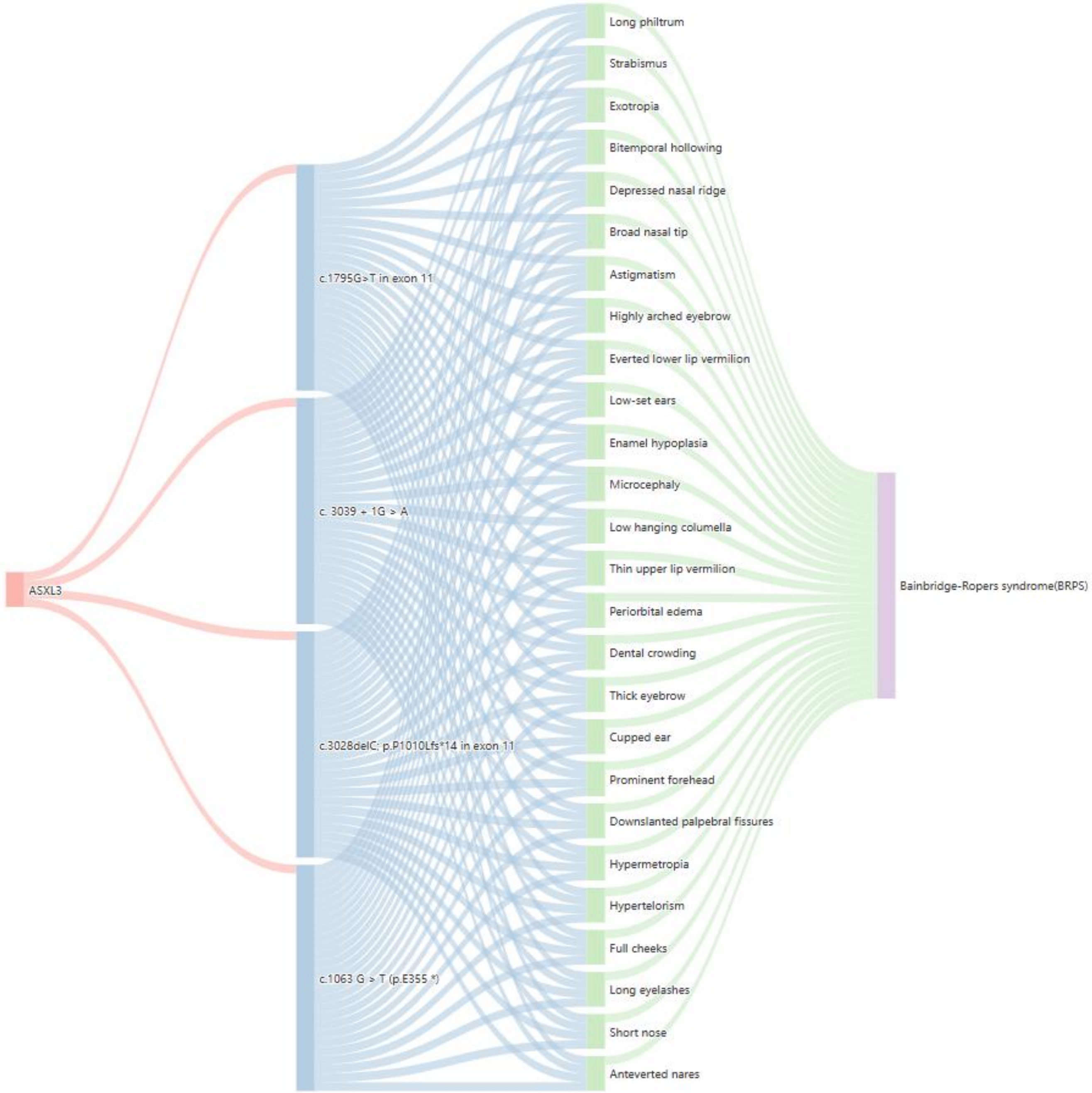

Supplementary Figure 1. Sankey diagram analysis of ASXL3. The diagram is structured into four layers, starting with the ASXL3 gene in the first layer, followed by variant details in the second layer. The third layer displays associated facial phenotypes, and the fourth layer details rare genetic diseases linked to these variants.

Supplementary Figure 2 shows the Sankey diagram analysis of COL27A1(URL).

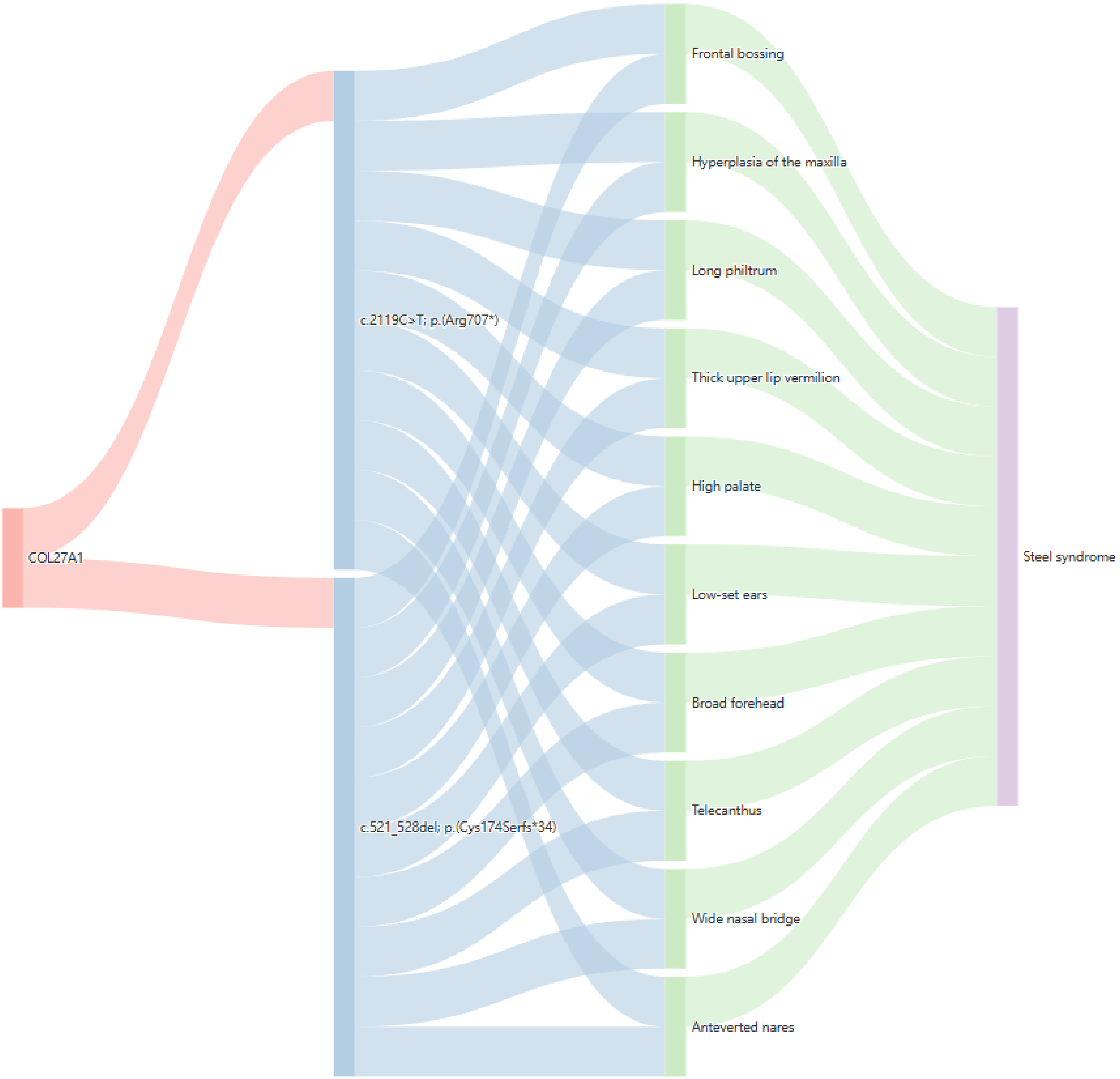

Supplementary Figure 2. Sankey diagram analysis of COL27A1. The diagram is structured into four layers, starting with the COL27A1 gene in the first layer, followed by variant details in the second layer. The third layer displays associated facial phenotypes, and the fourth layer details rare genetic diseases linked to these variants.

Supplementary Figure 3 shows the Sankey diagram analysis of SURF1(URL).

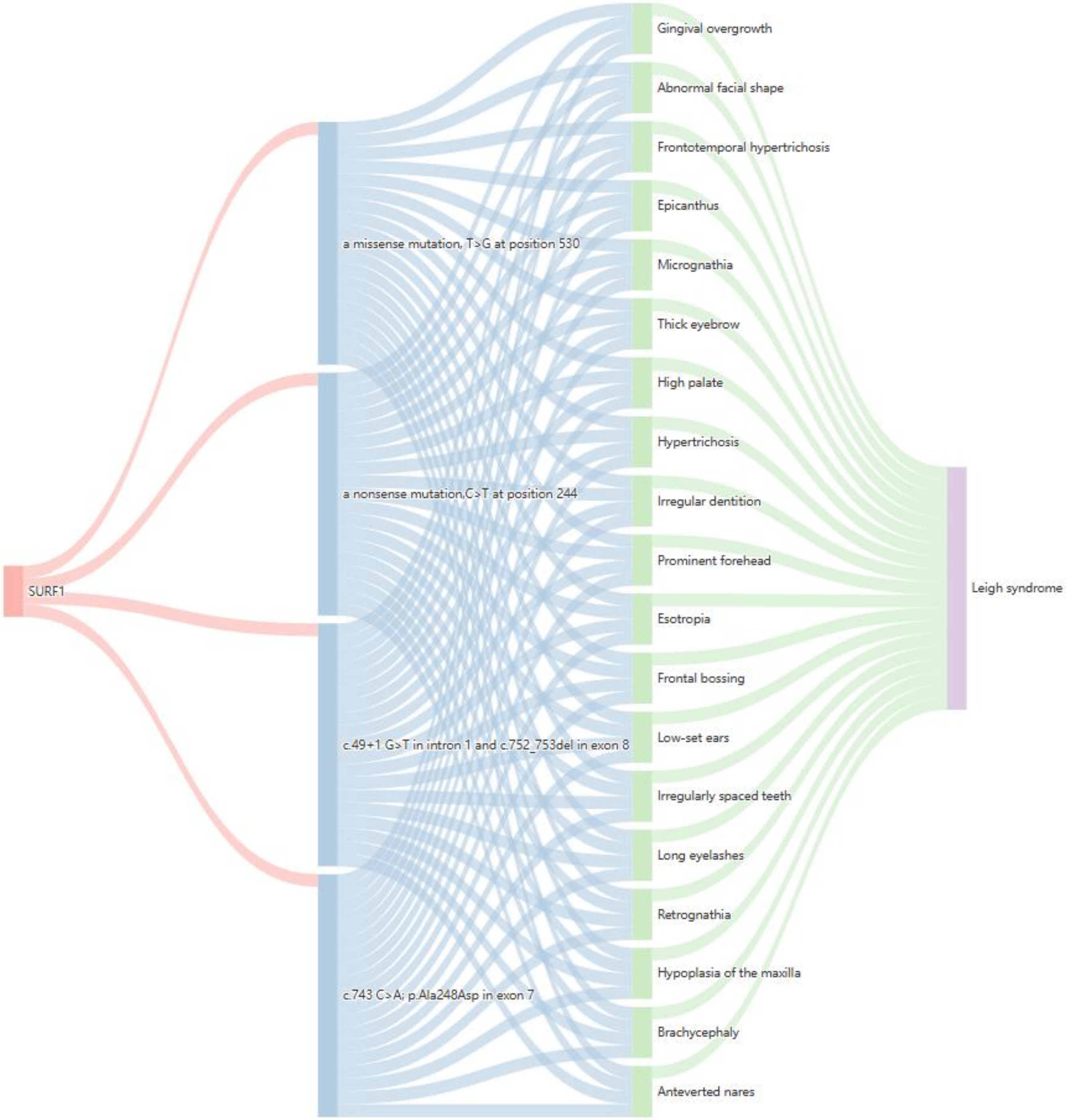

Supplementary Figure 3. Sankey diagram analysis of SURF1. The diagram is structured into four layers, starting with the SURF1 gene in the first layer, followed by variant details in the second layer. The third layer displays associated facial phenotypes, and the fourth layer details rare genetic diseases linked to these variants.

Supplementary Figure 4 shows the Sankey diagram analysis of KRAS(URL).

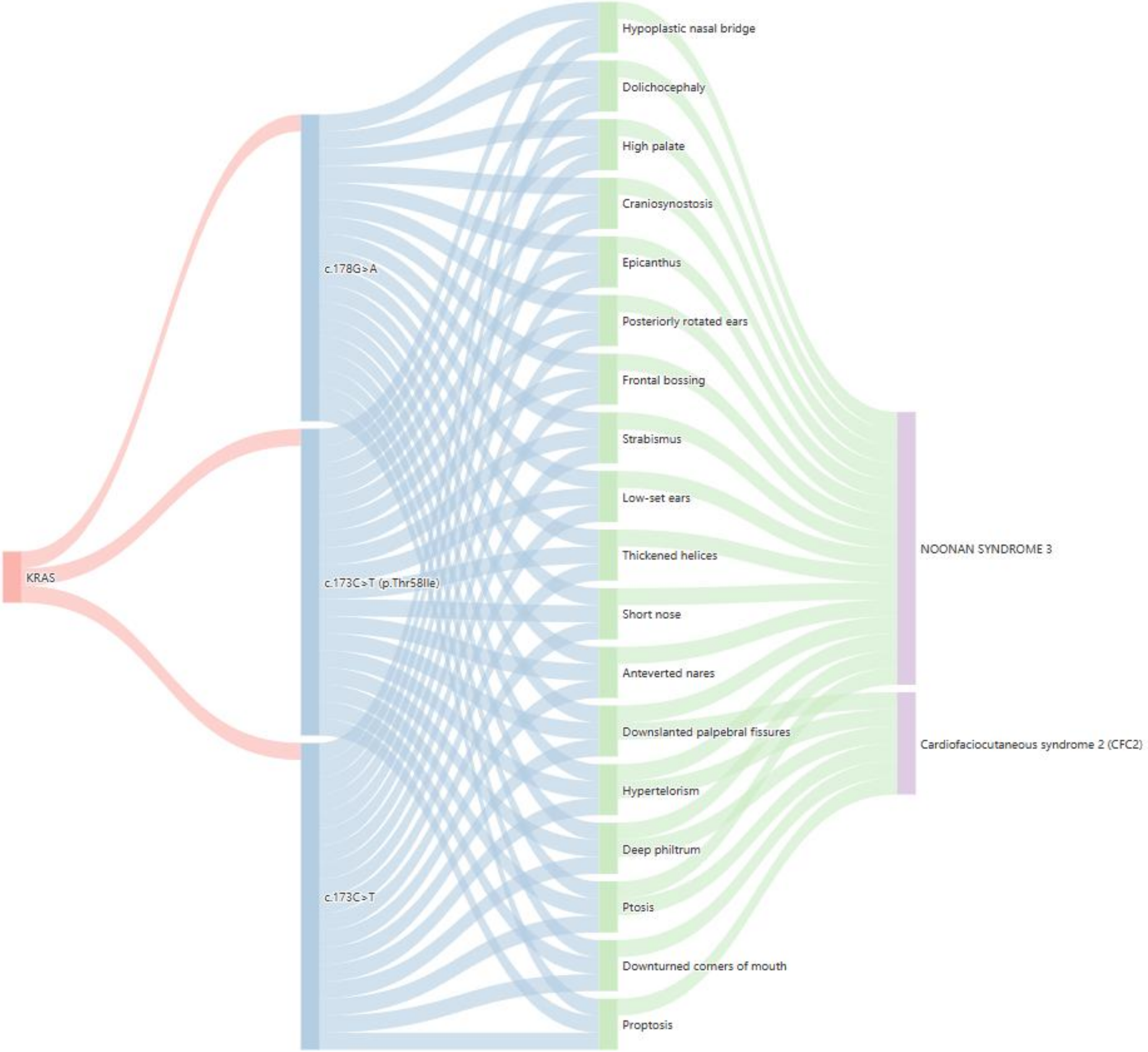

Supplementary Figure 4. Sankey diagram analysis of KRAS. The diagram is structured into four layers, starting with the KRAS gene in the first layer, followed by variant details in the second layer. The third layer displays associated facial phenotypes, and the fourth layer details rare genetic diseases linked to these variants.
